# Burns and Scalds

**Published:** 1880-08

**Authors:** 


Burns and Scalds.—Dr. George F. Shrady treats burns very successfully with a dressing made as follows:
℞ Gum Acacia, ℥ iii; Gum Tragacanth, ʒ i; Carbolized Water, 1-60 1 pint, and molasses ʒ i, mix. It is applied to the burnt surface with a broad, flat camel's hair brush immediately after the occurrence of the accident. It dries in the course of an hour or two, and may be renewed at proper intervals to form an unyielding pellicle or covering to the denuded and exposed surface. It is stated that generally four applications are sufficient to form the scab or coating for the surface. This artificial covering effectually excludes the air with whatever irritating qualities it may be impregnated, and it can be punctured or elevated to allow of the escape of pus should it be found, or accumulate. The pellicle cracks and peels off in the course of a fortnight, leaving the surface in a healthy or granulating condition, which is to be treated with zinc ointment, or other proper dressing. Covering the denuded surface with a mixture of white lead and linseed oil, as recommended by Prof, S. D. Gross some years ago, is also a very comforting and effective method of relieving accidents from fire, scalding water or vapor. The *Allgemeine Wiener Zietung* extolls the oil of peppermint as the best of known agent in the treatment of burns and scalds. It is sometimes advisable to dilute it one half with glycerine before its application.

